# The effect of non-communicative eye movements on joint attention

**DOI:** 10.1177/1747021820945604

**Published:** 2020-08-05

**Authors:** Nathan Caruana, Ayeh Alhasan, Kirilee Wagner, David M Kaplan, Alexandra Woolgar, Genevieve McArthur

**Affiliations:** 1 Department of Cognitive Science, Macquarie University, Sydney, New South Wales, Australia; 2 Perception in Action Research Centre, Macquarie University, Sydney, New South Wales, Australia; 3 MRC Cognition and Brain Sciences Unit, University of Cambridge, Cambridge, UK

**Keywords:** Joint attention, eye tracking, eye gaze, social interaction, social cognition

## Abstract

Eye movements provide important signals for joint attention. However, those eye movements that indicate bids for joint attention often occur among non-communicative eye movements. This study investigated the influence of these non-communicative eye movements on subsequent joint attention responsivity. Participants played an interactive game with an avatar which required both players to search for a visual target on a screen. The player who discovered the target used their eyes to initiate joint attention. We compared participants’ saccadic reaction times (SRTs) to the avatar’s joint attention bids when they were preceded by non-communicative eye movements that predicted the location of the target (Predictive Search), did not predict the location of the target (Random Search), and when there were no non-communicative eye gaze movements prior to joint attention (No Search). We also included a control condition in which participants completed the same task, but responded to a dynamic arrow stimulus instead of the avatar’s eye movements. For both *eye* and *arrow* conditions, participants had slower SRTs in Random Search trials than No Search and Predictive Search trials. However, these effects were smaller for eyes than for arrows. These data suggest that joint attention responsivity for eyes is relatively stable to the presence and predictability of spatial information conveyed by non-communicative gaze. Contrastingly, random sequences of dynamic arrows had a much more disruptive impact on subsequent responsivity compared with predictive arrow sequences. This may reflect specialised social mechanisms and expertise for selectively responding to communicative eye gaze cues during dynamic interactions, which is likely facilitated by the integration of ostensive eye contact cues.

Successfully navigating social interactions depends on a range of cognitive abilities, including joint attention which is the process by which we coordinate attention to share information with others. Joint attention involves one person initiating a joint attention bid by intentionally cueing the location of an object or event (e.g., by looking, pointing, or naming), and a second person responding to that bid by shifting their attention to the cued location. However, communicative eye movements which signal a joint attention bid are usually embedded in the context of other non-communicative gaze shifts (e.g., incidentally looking at other objects). As such, successfully responding to a gaze-cued joint attention bid relies on the ability to evaluate multiple cues and then selectively respond to those most likely to be intentional and communicative (Bruinsma et al., 2004; Caruana et al., 2017a). This ability begins to emerge in the first year of life and is pivotal for the development of language and our ability to make inferences about others’ thoughts, intentions, and beliefs (i.e., mentalising; Adamson et al., 2009; Charman, 2003; Dawson et al., 2004; Mundy et al., 1990; Murray et al., 2008; Tomasello, 1995).

Although the importance of joint attention to development is well established (see Mundy, 2018, for a review), we know relatively little about the cognitive mechanisms that support this social-cognitive ability. In particular, almost all interactive paradigms that examine joint attention behaviour (e.g., Redcay et al., 2010; Saito et al., 2010; Schilbach et al., 2010; Oberwelland et al., 2016), as well as the non-interactive paradigms traditionally used in this field (see Caruana, McArthur, Woolgar, & Brock, 2017b, for review), only ever required participants to evaluate a single, obvious, gaze shift. In the real world, by contrast, the communicative value of a person’s eye gaze is often subtly conveyed and can be unpredictable.

This unpredictability of real-world eye gaze arises, in part, because communicative eye gaze cues occur among non-communicative gaze cues, such as glancing away from a social interlocutor mid-conversation while listening or thinking. Such non-communicative eye movements, while providing some information about the attention and perspective of a social partner, are often not useful for predicting upcoming joint attention opportunities or their locus in the immediate environment. We know that humans—from a very young age—are able to discriminate communicative eye movements from such non-communicative eye movements to successfully engage in joint attention. At 14 months of age, infants typically demonstrate an ability to preferentially follow an interactive partner’s gaze shifts after their partner establishes eye contact with them, compared with other non-communicative eye gaze shifts or head turns (Farroni et al., 2003; Senju & Csibra, 2008). However, the cognitive mechanisms supporting this ability are not well understood.

In this study, we sought to redress this knowledge gap by studying the effect of non-communicative gaze on joint attention responsivity. We used an interactive experimental paradigm that we have previously used to investigate the cognitive and neural aspects of joint attention (Caruana et al., 2015; Caruana, McArthur, Woolgar, & Brock, 2017; Caruana, Spirou & Brock, 2017). Of critical importance to this study, this paradigm—unlike non-interactive gaze cueing paradigms—provides a dynamic mix of non-communicative and communicative eye gaze behaviours which participants must continuously evaluate and intentionally respond to in order to coordinate with their social partner (see Caruana, McArthur, Woolgar, & Brock, 2017, for an in-depth discussion and review of this approach). For this study, we adapted this paradigm to manipulate the presence and predictability of non-communicative eye gaze behaviour in the lead-up to a joint attention episode. In a cooperative game, participants were asked to initiate and respond to joint attention with an avatar whom they were told was being controlled by a person called “Alan,” but was actually controlled by a gaze-contingent algorithm. In an initial “search phase,” the participant and Alan each searched a different row of three houses on a computer screen for a burglar (see Figure 1). Each time they fixated a house, its door opened to reveal the burglar or an empty space. In half the trials, participants found the burglar, made eye contact with Alan, and then guided him to the burglar by looking at the correct house. These were joint-attention *initiator* trials. In the remaining trials, the participant discovered that all their allotted houses were empty and waited until Alan finished searching his own houses. Alan then established eye contact with the participant and guided them to the house concealing the burglar. These were joint-attention *responder* trials. Importantly, our gaze-contingent algorithm ensures that Alan completes his search after the participant, so that once the participant fixates on Alan’s face, he searches 1–2 more houses before establishing eye contact. This ensured that participants attend to Alan’s non-communicative and communicative eye gaze behaviours and allowed us to manipulate the presence and predictability of non-communicate gaze cues. In addition, as the cognitive mechanisms for dealing with non-communicative gaze will involve social (e.g., evaluating your social partner’s intention), visual (e.g., processing the visual information), and attention processes (e.g., suppressing unnecessary attention shifts to irrelevant spatial cues), we included a non-social condition in which participants complete the same task with dynamic arrow cues instead of eye gaze cues.

**Figure 1.**
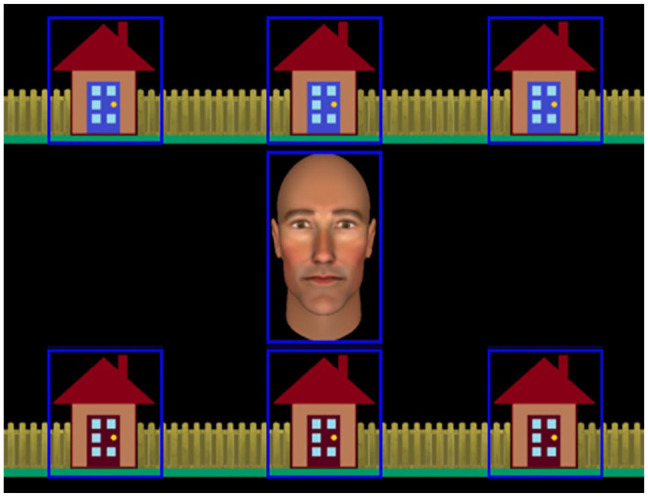
Stimulus used in the interactive joint attention task, including the central avatar and the six houses in which the burglar could be hiding. Gaze-related areas of interest (AOIs) are represented by blue rectangles. These were not visible to participants.

In five previous studies (Caruana et al., 2015, 2018, 2019; Caruana, McArthur, Woolgar, & Brock, 2017; Caruana, Spirou & Brock, 2017), we found that participants’ saccadic reaction times (SRTs) were significantly *slower* to Alan’s gaze cues than to arrows. By contrast, non-interactive studies that present only a single, and thus, unambiguous gaze cue report more *rapid* cueing for social gaze compared with non-social cues (see Frischen et al., 2007, for review). This suggests that the non-communicative cues in our gaze condition may have slowed our participants down. Indeed, when we directly compared trials that did and did not include non-communicative gaze cues (see Caruana et al., 2017a, 2019), participants were slower in the presence of non-predictive cues. We have suggested that slower responses may be due to the additional processing time required to evaluate and selectively respond to the avatar’s communicative eye movements. However, in this previous work, it was not possible to rule out the contribution of the extra information that needed to be disregarded *in general* in the eye condition, because the arrow control condition did not include any “non-communicative” arrows. That is, the arrow did not “search” for the burglar and only appeared after the participant completed their search and fixated a central fixation point. This was an intentional design consideration for the non-social condition, to avoid the possibility of a searching arrow generating a perceived sense of agency, and evoking a social response. Consequently, we currently do not know if the influence of non-communicative eye movements on joint attention reflects a uniquely social phenomenon or alternatively, a visual or domain general process. Furthermore, within social contexts, we do not yet understand how non-communicative eye movements influence joint attention, beyond the fact that they do. For example, is it the case that all types of non-communicative eye movements are harmful to joint attention responses? Or could some types of non-communicative eye movements actually enhance the ability to respond to joint attention bids?

This study addressed these questions in two experiments. In Experiment 1, we compared participants’ SRTs to an avatar’s successful bid for joint attention in four conditions: when the avatar made no non-communicative eye movements prior to making a bid for joint attention (No Search), when the avatar made non-predictive (i.e., random) eye movements before initiating joint attention (Random Search), and in two equivalent non-social conditions using arrows instead of eye movements. We predicted that SRTs would be significantly slower in the Random Search task than the No Search task because of the extra processing required to discriminate communicative from non-communicative eye movements. We further predicted that if this effect is a social phenomenon, it would be larger in the social (eye gaze) condition than in the non-social (arrow) condition.

In Experiment 2, we asked whether the nature of the non-communicative cues matter. We compared Random Search to a condition in which the avatar’s eye gaze always searched the correct burglar location just before making eye contact and initiating joint attention (Predictive Search). These conditions were closely matched in the complexity of the spatial information presented to participants, but differed in their predictiveness of the target location. In our matched non-social (i.e., arrow) conditions, arrow stimuli behaved in the exact same way as the avatar’s eye gaze. As this has never been investigated before, we were unable to make an evidence-based prediction. However, we reasoned that SRTs should be faster in Predictive Search trials than in Random Search trials because participants would be provided with spatial information that reliably predicts the target location. We also reasoned that this effect would be larger for social than non-social trials because participants would be more likely to engage in mentalising mechanisms (e.g., perspective taking or representing the intentions of their partner), and therefore make predictions, during the social condition. For example, they might predict that the location that the avatar looked at last during his search might indicate the burglar’s location (assuming he terminates his search once finding the burglar).

## Methods

### Ethics statement

All procedures implemented in this study were approved by the Macquarie University Human Research Ethics Committee.

### Participants

Participants were recruited into the two experiments from undergraduate psychology units at Macquarie University and were given course credit for their time. All participants had normal or corrected-to-normal vision and had no history of neurological injury or impairment. Thirty-five participants were recruited for Experiment 1. Nine participants were excluded due to failed eye-tracking calibration (*n* = 2), rejection of the deceptive cover story (*n* = 6), or excessive trial loss due to errors (more than 2 *SD*s above group average; *n* = 1). Thus, the final sample in Experiment 1 comprised 26 participants (*M*_age_ = 19.92, *SD* = 3.01, 5 males, 21 females). Thirty-four participants were recruited for Experiment 2. Six participants were excluded as they rejected the deceptive cover story (*n* = 5) or made excessive errors (*n* = 1). Thus, there were 28 participants in the final sample for Experiment 2 (*M*_age_ = 21.25, *SD* = 6.36, 7 males, 21 females).

### Design and procedure

In each experiment, we recorded participant eye movements (accuracy and SRTs) in four conditions: two *context* conditions (Experiment 1: No Search vs Random Search; Experiment 2: Random Search vs Predictive Search) and two *stimulus* conditions (eyes vs arrows; see below for details of these conditions). Participants completed four blocks of trials (one for each condition), which each comprised 30 responding trials (the focus of the experiments) and 30 initiating trials (included to establish the necessary reciprocal task context). The order of conditions was counterbalanced across participants. The eye gaze and arrow versions of each search condition were always administered consecutively to minimise switching between search contexts (e.g., Eyes Random Search, Arrow Random Search, Eyes No Search, Arrow No Search). Within each block, trial order was randomised to ensure that the location of the burglar, the location of blue doors, and the number of gaze shifts made by the avatar were not conflated with order effects. The experimental stimuli were presented using Experiment Builder 1.10.165 (SR Research Ltd., 2004, Ontario, Canada).

Participants’ eye movements were recorded from the right eye with a sampling rate of 500 Hz. Stimuli were presented on a 27-inch AOC monitor positioned 85 cm away from the participant. We used a remote desktop-mounted EyeLink 1000 (SR Research Ltd., Ontario, Canada) to record eye movements. Head movements were stabilised with a chin-rest and a 9-point eye-tracking calibration was implemented before each block. Seven gaze-related areas of interest (AOIs; see Figure 1) over the houses and avatar stimulus were used by our gaze-contingent algorithm and for subsequent analyses (see Caruana et al., 2015, for detailed description).

Accuracy was calculated as the proportion of trials in which the participant succeeded in catching the burglar, excluding trials in which the experiment was paused to recalibrate the eye tracker or trials that included an error. There were three types of errors on responding trials: *location errors*, which occurred when a participant fixated on the wrong house; *time-out errors*, which occurred when it took a participant over 3 s to respond; and s*earch errors*, which occurred when a participant spent more than 3 s looking away from the avatar or the houses before establishing eye contact. Location and time-out errors triggered the appearance of a red burglar in the correct location. Search errors triggered the presentation of “Failed Search” in red text between the two rows of houses.

**Figure 2.**
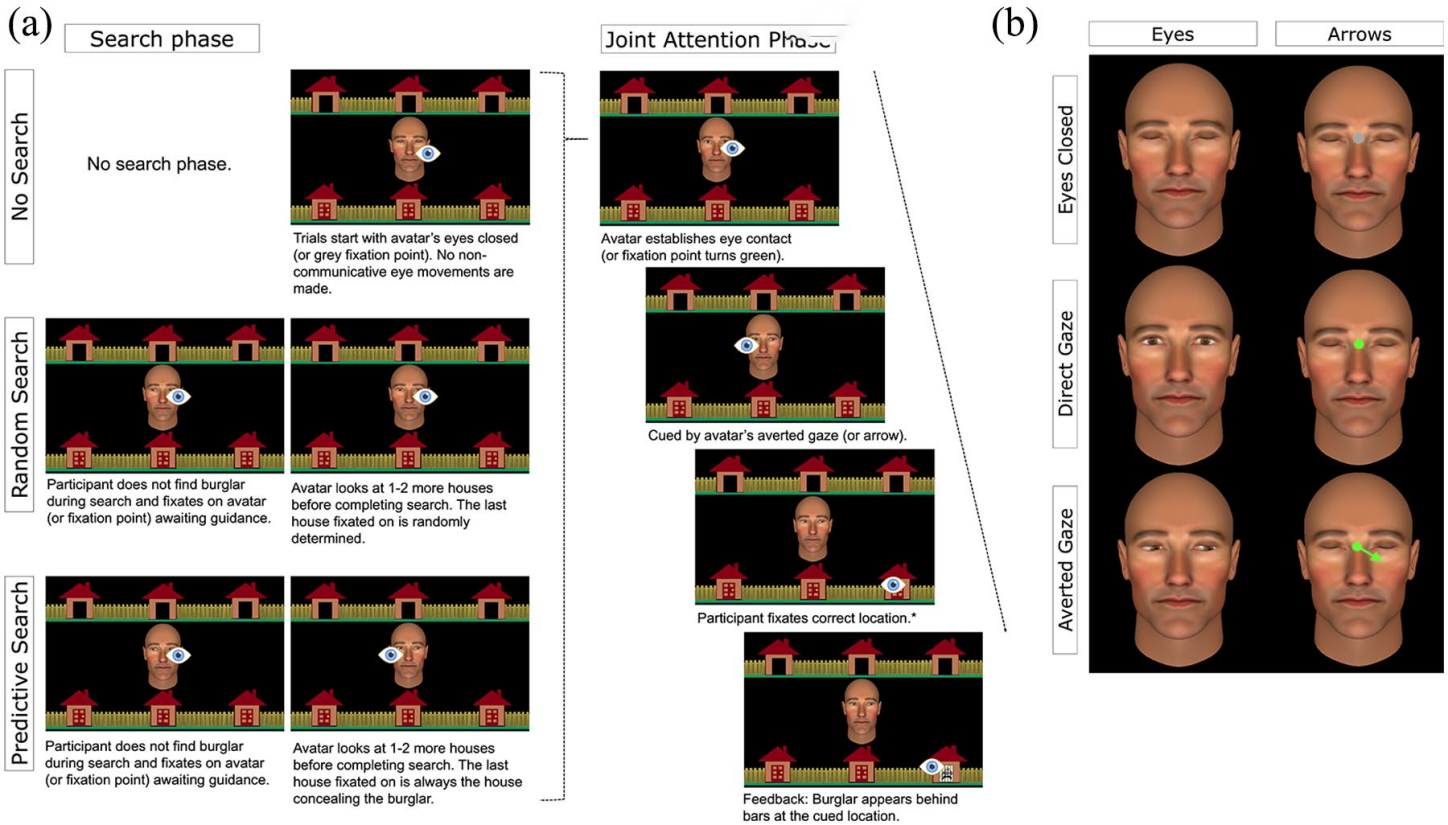
(a) Schematic representation of the trial sequence by task using social eye gaze stimulus. The schematic eye icon represents the fixation location required from participants at each point in the trial (i.e., this was not part of the task stimulus visible to participants). *SRT is measured as the latency between cue presentation and onset of responsive saccades. (b) Example central stimulus by condition representing the analogous non-social stimulus used in the arrow condition for eyes closed, direct gaze, and averted gaze. The background stimulus and the timing of stimulus presentation were identical across stimulus conditions (Eyes, Arrows).

To calculate SRTs, we first removed error trials or anticipatory saccades (SRTs < 150 ms). For the remaining trials, we measured the number of milliseconds between the presentation of the avatar’s eye gaze cue or the arrow cue and the onset of the participant’s responding saccade towards the correct burglar location (see Figure 2).

### Conditions

Participants in both experiments played a cooperative game with an on-screen avatar—an anthropomorphic face that subtended 6.08° × 3.65° of visual angle—believed to be controlled by the eye movements of a real person, in the adjacent laboratory. Participants were told the other person was a member of the research team, named Alan. In fact, the avatar’s eye-movements were controlled by a gaze-contingent algorithm (see Caruana et al., 2015, for a task and video demonstration). Participants cooperated with the avatar to catch a burglar hiding inside one of the six houses presented on the screen, each subtending 3.58° of visual angle. Participants were always required to monitor the upper row of houses with blue doors, while the avatar searched the lower row of houses with red doors. On half the trials, participants found the burglar and were required to make eye contact and guide the avatar to the correct location (i.e., initiating trials). On the remaining half of trials, the participants found all their houses to be empty and hence were required to wait for the avatar to guide them to the correct location. Although both responding and initiating trials were necessary to establish a realistic, collaborative, and reciprocal task context, as mentioned above, the current analyses focus exclusively on responding trials.

#### Context conditions

##### Random search condition (Experiments 1 and 2)

Random Search trials began with a “search phase” in which participants were required to search through the upper row of houses with blue doors while Alan searched the lower row of houses with red doors. Participants could only see the contents of their allocated row of houses and could not see inside Alan’s houses. Participants searched their houses by fixating the houses in any order. Once fixated, the door opened to reveal an empty house or the burglar. At the same time, Alan’s gaze would shift to “search” his own allotted houses in a randomised order. We systematically varied across trials whether 0–2 of the participant’s houses were already opened and empty at the beginning of the trial. This introduced variability in the spatial sequence of participants’ search behaviour and helped justify Alan’s spatially unpredictable search behaviour. Whoever found the burglar was required to guide the other person to the burglar’s location by initiating joint attention, and the other person was required to respond appropriately.

On responding trials, participants discovered that all of their allotted houses were empty (Figure 2). Once the participant fixated back on the avatar’s face, Alan searched 1–2 more houses before making eye contact. Critically, the last house Alan looked at before making eye contact was randomly determined and was not predictive of the burglar’s location. Upon establishing eye contact, Alan averted his gaze towards the correct house to initiate joint attention. Participants were required to respond by fixating the cued house. Once joint attention was achieved, the burglar appeared behind bars at the correct location to provide positive feedback.

##### No search condition (Experiment 1)

This condition removed the avatar’s search behaviour from each trial. At the start of each trial, the participant could see the avatar with his eyes closed and one of two door arrangements. On initiating trials, they could see one blue door, which they were told concealed the burglar, and on responding trials they could see all houses opened and empty. Participants were required to establish eye contact with the avatar at the beginning of the trial. The avatar’s eyes opened 500–1,000 ms (jittered with a uniform distribution) after the participant fixated on the eye region. On initiating trials, participants simply needed to make a single gaze shift to the visible blue door to initiate joint attention. On responding trials, after establishing eye contact, the avatar made a single gaze shift towards the burglar’s location after a further 500–1,000 ms, which participants had to follow.

##### Predictive search condition (Experiment 2)

In this condition, participants were told that the avatar—although identical in appearance to the avatar in the other eye gaze conditions—was now being controlled by a different member of the research team named “Tony.” This task was the same as the Random Search condition except that, unlike Alan, Tony’s final gaze shift during the search phase was always directed to the burglar’s location and was thus predictive of his subsequent joint attention bid following the establishment of eye contact. Participants were told that they were interacting with different members of the research team during the two tasks to provide a plausible, yet implicit, explanation for the systematic differences in the avatar’s behaviour across the two task blocks. Critically, participants were not explicitly told about the task manipulation.

#### Stimulus conditions

For each context condition described above (Random Search, Predictive Search, No Search), we implemented control trials matched on non-social task demands (e.g., attentional, oculomotor, and inhibitory control) which were completed as separate blocks. This resulted in two stimulus conditions (Eyes, Arrow). In the arrow conditions, participants were told that they would complete the task with the assistance of a computer-controlled arrow stimulus. To match the visual context between stimulus conditions, the avatar’s face remained on the screen with his eyes closed on arrow trials. At the beginning of the trial, a grey fixation point subtending 0.29° of visual angle was presented in between the avatar’s eyes (analogous to closed eyes only on social eye gaze trials). This fixation point turned green (analogous to direct gaze on social eye gaze trials) and was then replaced by a central green arrow subtending 1.08° of visual angle (analogous to averted gaze on social eye gaze trials) which updated during the search phase (on Random and Predictive Search trials) to point at a different house each time the participant searched a new location. Once the participant completed their search and fixated back within the central AOI, the arrow stimulus updated 1–2 more times, pointing at a different house each time, before being replaced by the green fixation point. Thus, unlike previous studies implementing this paradigm, the arrow stimulus was precisely programmed to mimic the avatar’s “searching” gaze behaviour (Figure 2). Following the search phase on Random and Predictive Search trials, or at the beginning of the trial on No Search trials, the green fixation point was replaced by a single green arrow which pointed towards the target house. Again, the algorithm driving stimulus presentation in the eyes and arrow conditions was identical, for both responding and initiating trials. The arrow conditions only differed from the eye gaze conditions in terms of (1) the stimuli presented to participants (i.e., eyes vs arrows) and (2) whether participants believed the stimuli to be human- or computer-controlled.

### Subjective ratings

For the aforementioned conditions to be valid, it was important that participants were convinced that Alan and Tony were real people controlling the on-screen avatar. We therefore asked participants to use a scale of 1 (*not at all*) to 10 (*extremely*) to rate their experience of how: (1) difficult, natural, and pleasant they found each block; (2) cooperative they found their partner on eye gaze blocks; (3) “human-like” the interaction felt; and (4) human-like the avatar’s behaviour seemed. Participants were also asked to indicate the degree to which they preferred: (1) interacting with a virtual avatar to real humans, (2) the No Search or Predictive Search conditions to the Random Search condition, and (3) the arrow conditions to the eye gaze conditions. Once the responses were recorded, participants were debriefed, and asked to rate how convinced they were that they had been interacting with a real person (1 to 10 = *completely unconvinced* to *convinced*). Only participants whose rating was 6 or above were included in the analysis. Finally, participants reconsented to participate in the study.

Given that the context condition manipulation in Experiment 2 (Random vs Predictive Search) was more subtle than that in Experiment 1 (Random vs No Search), we were also interested in the extent to which participants in Experiment 2 were aware of the condition manipulation. As such, prior to being debriefed about the deceptive cover story, participants in Experiment 2 were additionally asked whether they noticed any systematic differences between the Random and Predictive Search conditions for both the eye gaze and arrow blocks. Out of the 34 participants, 15 reported noticed differences in Alan and Tony’s behaviour on eye gaze blocks, but only 8 noticed differences between the matched tasks on arrow blocks. A comprehensive summary of subjective ratings data for both experiments is reported in Supplementary Material 1.

### Statistical analyses

The accuracy and SRT data (i.e., using interest area and trial reports) were exported using DataViewer software (SR Research Ltd., Ontario, Canada) and then screened and analysed in R. All raw data, R code (with annotated descriptions) and analysis output are available at the Open Science Framework (https://osf.io/jb8fv/). Statistical analyses of logistic and linear mixed random effects were conducted for accuracy and SRT data, respectively, to test for context and stimulus interaction effects. These analyses were implemented using the maximum likelihood estimation method within the *lme4* R package (Bates, 2005), and *p*-values were estimated using the *lmerTest* package (Kuznetsova et al., 2015). We implemented mixed random-effects models rather than traditional analyses of variance (ANOVAs) because mixed random-effects models can account for both subject and item-level variance (i.e., random effects) when estimating fixed effects and interactions. Unlike traditional ANOVAs on aggregated means, mixed random-effects analyses are also robust to missing data and are suitable for datasets with unbalanced observations in each condition because each trial, rather than each subject, is treated as a unique observation (Quené & van den Bergh, 2004, 2008). However, we also included traditional ANOVA in the accompanying R code and output to enable easy comparisons between these analyses.

Fixed factors (i.e., task and condition) were treated as summed contrasts. In line with recommendations for implementing mixed random-effects models, we initially defined models with maximally defined random-factor structures, with random intercepts for trial and by-subject random slopes for the fixed effects and associated interactions (Barr et al., 2013). Due to the complexity of our random-effects structure, models with random slopes for the interaction effect could not be estimated (Barr et al., 2013). Hence, we defined our models with the most maximal yet parsimonious random-effects structure afforded by our data. For the accuracy model, this resulted in a model including random intercepts for subject and trial. For the SRT model, this included by-subjects random intercepts and slopes for the effect of condition and task as well as random intercepts for trial.^1^

For SRT analyses, data were transformed using an inverse transformation because the residuals of the raw data were found to violate the normality assumption (see Balota et al., 2013). We have included the output for SRT analyses on transformed and untransformed data (including Q-Q normality plots) in the accompanying output. Analyses on transformed SRT data are reported below. Post hoc contrasts were also tested using emmeans package (Lenth et al., 2019) to investigate the context by stimulus interaction effect further. All analyses had a significance criterion of *p* < .05, except for the follow-up post hoc contrasts, where we implemented a Bonferroni correction on the four follow-up comparisons (α = .0125).

In line with our previous analyses (see Caruana et al., 2019), we compared a series of mixed random-effects models using chi-square likelihood ratios to quantify the variance explained by each of our fixed effect and interactions parameters. These analyses were conducted in lieu of traditional effect-size measures, which are unable to account for the variance explained by each fixed effect, over and above variance already explained by the defined random effects. First, we defined a model containing only the maximally defined random effects structure used in the accuracy and SRT analyses (i.e., without including fixed effect factors). Then we defined a series of models by adding one of our fixed-effect parameters at a time (see accompanying R code for a detailed description). The resulting chi-square likelihood ratios indicated the extent to which each parameter improved the model’s fit.

## Results

### Experiment 1

#### Accuracy

**Figure 3.**
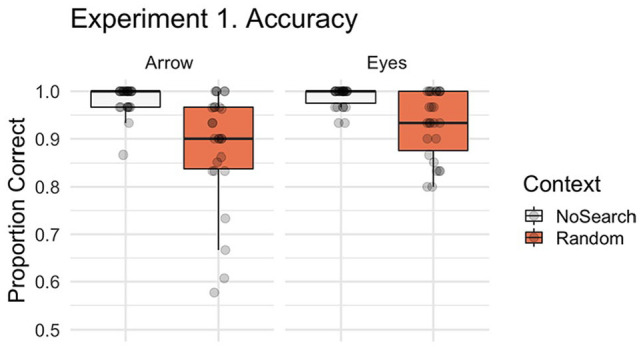
Boxplot with individual data points illustrating the proportion of correct trials by context (Random Search, No Search) and stimulus (i.e., social, non-social). In all boxplot figures, whiskers extend (as in a conventional Tukey’s boxplot) 1.5 times the length of the box (i.e., the interquartile range of the 1st and 3rd quartiles). For accuracy in Experiment 1 we identified a significant context effect, characterised by more errors following the Random Search than No Search.

First, we asked whether the presence of non-predictive spatial signals differentially affected participants’ ability to correctly respond to subsequent eye gaze and arrow cues. Figure 3 illustrates the accuracy data for the context and stimulus conditions for Experiment 1. Participants made significantly more errors in the Random than the No Search trials (main effect of context, β = 2.017, *SE* = 0.231, *z* *=* 8.740, *p* < .001) irrespective of stimulus. There was no significant stimulus effect (β = −0.473, *SE* = 0.260, *z* *=* −1.817, *p* = .069) or context by stimulus interaction (β = 0.215, *SE* = 0.460, *z* *=* 0.467, *p* = .641). The majority of errors were Location errors (*M* = 4.83% of trials, *SD* = 3.70) with less than 1% of trials being classified as a Search (*M* = 0.58, *SD* = 1.02) or Time-out errors (*M* = 0.35, *SD* = 0.71).

#### SRT

**Figure 4.**
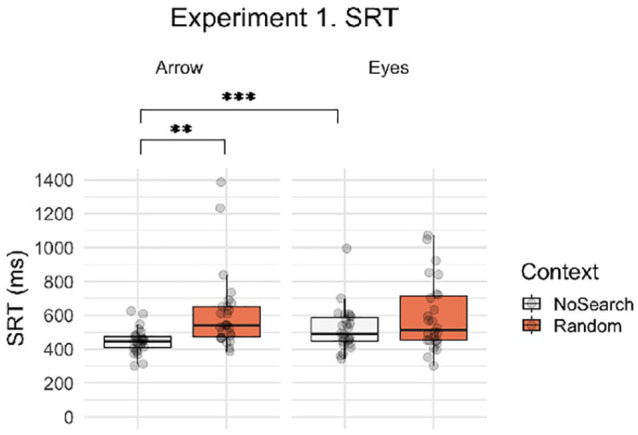
Boxplots with individual data points for SRTs by context (Random Search, No Search) and stimulus (i.e., social, non-social). Asterisks indicate significant post hoc contrasts with Bonferroni correction (**p < .01, ***p < .001).

Next, we asked whether the presence of non-predictive spatial signals differentially influenced the speed with which participants were able to initiate saccadic eye movements in response to eye gaze and arrow cues. Figure 4 illustrates the SRT data for context and stimulus conditions for Experiment 1. Participants were significantly slower overall to respond during the Random Search than the No Search trials (main effect of context, β = 0.186, *SE* = 0.088, *t* *=* −2.107, *p* *=* .045) and were also significantly slower overall when responding to eyes than arrows (main effect of stimulus, β = 0.165, *SE* = 0.062, *t* *=* 2.651, *p* *=* .011). However, these main effects were modulated by a significant context by stimulus interaction (β = 0.254, *SE* = 0.054, *t* = 4.681, *p* < .001), which reflected a larger effect of context on arrows than on eyes. Post hoc contrasts revealed that there was a significant context effect in the arrow condition (β = 0.313, *SE* = 0.094, *t* *=* 3.319, *p* *=* .002)—in which participants were slower to respond to arrows in the Random Search than the No Search trials—but no significant effect of context for eyes (β = 0.059, *SE* = 0.094, *t* = 32.083, *p* = .534). In addition, there was a significant stimulus effect in the No Search condition—in which participants were significantly faster when responding to arrows than eyes (β = 0.292, *SE* = 0.068, *t* *=* 4.306, *p* < .001)*—*but not in the Search condition (β = 0.038, *SE* = 0.070, *t* *=* 0.544, *p* *=* .588). Therefore, these data indicate that the detrimental effect of an unpredictive spatial sequence on responsivity (i.e., context effect) was greater for arrows than for eyes. Mean SRT data are summarised by condition in Table 1.

**Table 1.** Saccadic reaction time *M* and *SD* by condition for Experiment 1.

Condition	Arrow no search	Arrow random	Eyes no search	Eyes random
*M* (*SD*)	446.87 (74.97)	613.19 (232.51)	519.92 (131.01)	593.00 (209.13)

Means and standard deviations are provided in the format *M* (*SD*).

#### Model fit analyses

To quantify the effects of stimulus and context, we compared improvement-in-model-fit as a function of each fixed effect parameter. Compared with the null model (i.e., no fixed-effect factors), adding the stimulus factor improved the model fit by 4.33 times, χ^2^(1) = 4.33, *p* = .037. Adding the context factor improved model fit a further 3.94 times, χ^2^(1) = 3.94, *p* = .047. While including the context factor to the null model first had no significant effect on model fit, χ^2^(1) = 0.998, *p* < .318, adding the stimulus effect to the context-only model significantly improved the model fit 7.27 times, χ^2^(1) = 7.27, *p* = .007. Critically, compared to a model containing fixed effect factors for both stimulus and context, adding the interaction parameter significantly improved the model fit by 21.77 times, χ^2^(1) = 21.77, *p* < .001. These analyses revealed a larger main effect of context than stimulus. However, the data also suggest that both factors explain unique variance in the data and that the data is best explained by a model which specifies a stimulus by context interaction.

### Experiment 2

#### Accuracy

**Figure 5.**
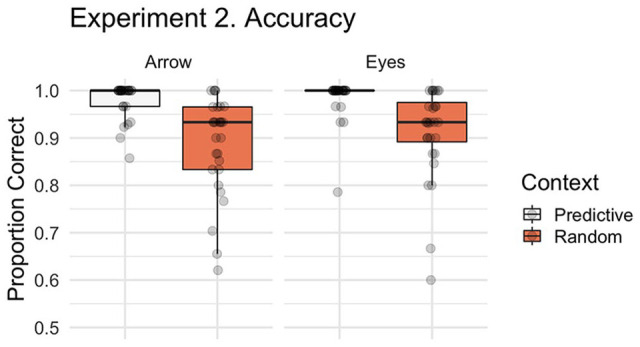
Boxplot with individual data points illustrating the proportion of correct trials by context (Random Search, Predictive Search) and stimulus (i.e., social, non-social). For accuracy in Experiment 2 we identified a significant context effect, characterised by more errors following the Random Search than No Search.

In Experiment 2, we examined whether participants’ ability to correctly respond to eye gaze and arrow cues were influenced by the predictiveness of the preceding pattern of eye gaze or arrow shift sequence. Figure 5 illustrates the accuracy data by stimulus and context for this Experiment. Participants made significantly more errors overall in the Random Search condition than the Predictive Search condition (main effect of context, β = 1.932, *SE* = 0.206, *z* *=* 9.366, *p* < .001). As in Experiment 1, we found no evidence of a significant stimulus effect (β = −0.301, *SE* = 0.220, *z* *=* −1.365, *p* = .172) or stimulus by context interaction (β = −0.023, *SE* = 0.412, *z* *=* −0.056, *p* = .956). Like Experiment 1, the majority of errors were Location errors (*M* = 4.88% of trials, *SD* = 3.91) with approximately 1% of trials being classified as a Search (*M* = 1.16, *SD* = 1.53) or Time-out error (*M* = 0.95, *SD* = 1.22).

#### SRT

**Figure 6.**
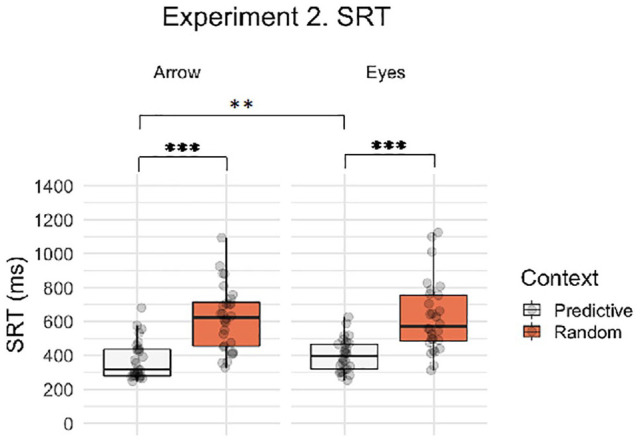
Boxplot with individual data points for saccadic reaction times on correct trials by context (Random Search, Predictive Search) and stimulus (i.e., social, non-social). Asterisks indicate significant post hoc contrasts with Bonferroni correction (**p < .01, ***p < .001).

Next, we asked whether saccadic response times to eye gaze and arrow cues were differentially influenced by the predictability of the preceding eye gaze or arrow spatial sequence. Figure 6 illustrates the SRT data for the context and stimulus factors in Experiment 2. Participants were significantly slower to respond on Random Search than Predictive Search trials (main effect of context, β = 0.890, *SE* = 0.088, *t* *=* 10.061, *p* *<* .001) and there was no main effect of stimulus (β = 0.132, *SE* = 0.084, *t* *=* 1.569, *p* *=* .126). However, there was a significant context by stimulus interaction (β = 0.217, *SE* = 0.073, *t* = 2.982, *p* = .003), reflecting a larger effect of context for arrows than eye stimuli. Post hoc contrasts showed a significant context effect for both arrow and eye stimuli separately (Arrow, β = 0.999, *SE* = 0.098, *t* *=* 10.175, *p* < .001; Eyes, β = 0.781, *SE* = 0.096, *t* *=* 8.110, *p* < .001). Furthermore, the stimulus effect was only significant in the Predictive Search condition, where participants were significantly faster to respond to arrows than eyes (β = 0.241, *SE* = 0.094, *t* *=* 2.567, *p* = .013), but not for the Random Search context (β = 0.024, *SE* = 0.092, *t* *=* 0.255, *p* = .800). Therefore, saccadic response times to eye gaze and arrow cues were differentially influenced by the predictability of the preceding eye gaze or arrow spatial sequence. Therefore, these data indicate that the predictive spatial sequence resulted in a larger response time advantage when cued by arrows than eyes. As in Experiment 1, response times to gaze cues appeared to be less affected by the preceding spatial sequence compared to the arrow condition. Mean SRT data are summarised by condition in Table 2.

**Table 2.** Saccadic reaction time *M* and *SD* by condition for Experiment 2.

Condition	Arrow predictive	Arrow random	Eyes predictive	Eyes random
*M* (*SD*)	372.13 (113.54)	620.45 (191.87)	401.83 (96.50)	626.86 (211.52)

Means and standard deviations are provided in the format *M* (*SD*).

#### Model fit analyses

To quantify fixed effects for the stimulus and context factors, we compared improvement in model fit as a function of each fixed-effect parameter. A model comprising the stimulus fixed-effect factor fit the data 4.07 times better than the null model, that is, no fixed effect factors; χ^2^(1) = 4.07, *p* = .044. Adding the context factor significantly improved the model fit by 41.98 times, χ^2^(1) = 41.98, *p* < .001. Including the context fixed-effect factor in the null model first significantly improved model fit 43.92 times, χ^2^(1) = 43.92, *p* < .001. Adding the stimulus effect did not significantly improve model fit, χ^2^(1) = 2.14, *p* = .143. Finally, compared to a model containing fixed-effect factors for both stimulus and context, adding the interaction parameter significantly improved the model by a further 8.85 times, χ^2^(1) = 8.85, *p* = .003. Again, these analyses reveal a larger main effect of context than stimulus, despite both factors explaining unique variance. The analyses also demonstrate that the data are best explained by a model that specifies a stimulus by context interaction.

## Discussion

Although gaze is an important cue for initiating and responding to joint attention, the communicative value of eye gaze can be ambiguous due to its dual function in signalling and sensing (Argyle & Cook, 1976; Gobel et al., 2015; Risko et al., 2016). On the contrary, eye gaze provides constant information about the perspectives of others which can be used to make predictions about their future communicative behaviours. As such, successfully responding to joint attention bids relies on the ability to evaluate eye movements *in context*, to differentiate those that are intended to “signal” rather than “sense” information. This study presents the first investigation of the influence that non-communicative eye movements have on our ability to respond to communicative gaze-cued joint attention bids. Specifically, we asked whether non-communicative eye movements hinder responsivity when they are non-predictive, whether they facilitate responsivity when they are predictive, and whether these effects were unique to eye gaze or reflected domain-general attention effects.

We implemented an interactive paradigm to compare responsive joint attention across two experiments comprising three contexts. For both eye gaze and carefully matched arrow stimuli, participants made significantly more errors when cues were embedded in a stream of non-communicative cues that were non-predictive (Random Search), compared with when non-communicative cues were either predictive (Predictive Search) or absent (No Search). This was also reflected in subjective ratings (see Supplementary material 1), which consistently reflected greater perceived difficulty during interactions in the Random Search context. These data suggest that while the presence of non-predictive spatial information can increase subjectively measured perceived difficulty and objectively measured response error, this does not appear to be a specifically social phenomenon, because it occurs for both eyes and arrow stimuli.

Of greater interest, there were three main findings from the SRT data. First, when cues were either presented in isolation (No Search) or could be predicted by the preceding context (Predictive Search), response times were faster for arrows than eyes, suggesting a fundamental advantage for responding to non-social arrow cues in unambiguous and predictable contexts. Second, participants were slower to respond to spatial cues (both arrows and eyes) when they were preceded by non-predictive spatial information (Random Search) compared to no information (No Search) or predictive information (Predictive Search), suggesting that these contexts have a global influence on responsivity. Third, and most importantly, the two effects interacted. In both studies, the response time “cost” of observing non-communicative *and* non-predictive cues (i.e., the context effect) was significantly smaller for eyes than arrows. This suggests that, compared to arrows, responsivity to communicative gaze cues is more stable across contexts and less affected by volatile and unpredictable (i.e., realistic) spatial information that may precede the joint attention cue.

### Slower responses to gaze cues in No Search and Predictive Search contexts

Despite using smaller arrow stimuli than our earlier work, we replicated our previous finding that participants are significantly faster to respond to arrows than eyes in a No Search context (see Caruana et al., 2017a). We also found the same pattern in our newly implemented Predictive Search context condition. The first possible explanation for this effect is that despite the additional measures taken to more closely match the perceptual salience of the gaze and arrow cues—our arrow stimuli may still be more visually salient and thus present a more potent spatial cue. However, this seems unlikely given that in psychophysics, the visual encoding of faces is consistently prioritised over non-face stimuli (e.g., Stein et al., 2012). There is also limited evidence from Posner-style cueing paradigms to suggest that arrows provide a more potent spatial cue than eyes, indeed there is some evidence for the contrary (see Itier & Batty, 2009, for review).

A second explanation is that the presence of direct gaze on social trials may interfere with subsequent responding in unambiguous contexts where there is either a single (No Search) or predictable (Predictive Search) gaze cue. One potentially important difference between our eye gaze and arrow cues was that the eye gaze cue was preceded by direct gaze (i.e., eye contact), whereas arrow cues were preceded by a green fixation point positioned between the avatar’s closed eyes (see Figure 2b). Eye contact is an ostensive signal which captures attention (Csibra & Gergely, 2009; Jarick et al., 2016; Senju & Hasegawa, 2005) and can induce psychophysiological arousal (Gale et al., 1972; Helminen et al., 2011; Klienke & Pohlen, 1971; Nicholls & Champness, 1971). As such, it is possible that the subjective experience of eye contact on social trials makes it harder for participants to disengage attention from the central stimulus and respond to the subsequent gaze shift which cues joint attention. Future research could probe this possibility further by implementing concurrent measures of arousal or attention, such as pupillometry, galvanic skin response, and event-related potential measures. However, given the highly dynamic nature of the visual stimulus in this task, this presents significant methodological challenges in obtaining reliable physiological measures.

A third, but related explanation is that our low-level sensitivities to direct gaze have downstream effects on the recruitment of higher-level cognitive processes such as mentalising (see Cañigueral & Hamilton, 2019, for a review). The recruitment of these parallel processes may attenuate the cognitive resources necessary for executing responses to subsequent gaze cues. We know from interocular suppression paradigms that faces and face-bound stimuli have privileged access to early, preconscious stages of visual processing (Stein et al., 2016) and that these effects are enhanced for faces with direct gaze compared with averted gaze (Stein et al., 2011). However, while this might result in the faster *detection* of direct gaze stimuli, it is also likely to trigger a cascade of higher-order representations which may contribute to *delayed responsivity* to subsequent eye movements. In line with both the “Fast-track Modulator” (Senju & Johnson, 2009) and “Watching Eyes” accounts (Conty et al., 2016), it is argued that the perception of direct gaze during genuine interactions rapidly and automatically activates subcortical pathways and substrates which initialise social-cognitive mechanisms for the representations of gaze direction (anterior superior temporal sulcus), intentions (posterior superior temporal sulcus, medial prefrontal cortex), and emotions (orbital frontal cortex, amygdala; Burra et al., 2019). The recruitment of these mechanisms might present a processing speed trade-off, which would result in slowed responses during unambiguous and predictable contexts where such additional processes present increased computational demand but at the same time have limited scope to facilitate responsivity. However, as discussed below, computing mental state representations might be more cost-effective during ambiguous social interactions where this can provide a template for differentiating communicative and non-communicative eye movements. Future work which manipulates the belief in whether the virtual partner is being controlled by another human or computer might help validate this potential interpretation by varying the extent to which mentalising mechanisms are recruited when people interact with the same stimuli (see Caruana, Spirou & Brock, 2017; Caruana & McArthur, 2017, 2019, for example).

### Relative stability in gaze-cued responses across contexts

In both experiments, we found that the advantage for arrow stimuli observed on No Search and Predictive Search trials was significantly attenuated on Random Search trials within the same individuals. These context-by-stimulus interactions, in both experiments, reflected a larger effect of context on response times to arrows than eyes. Moreover, for Experiment 1 the context effect (i.e., slower responses in the Random Search condition compared with the No Search condition) was only significant for arrows and not for eyes. This suggests that responsivity to gaze-cued joint attention bids is less affected by the presence of non-communicative eye movements in dynamic and unpredictable contexts (Random Search) compared with unambiguous (No Search) and predictable (Predictive Search) contexts, than responsivity to arrows. This relative stability in responding to communicative gaze information across contexts contrasts with the reliable advantage observed for arrows when they are presented in unambiguous and predictable spatial contexts rather than unpredictable contexts.

Together, the data across both experiments suggest that while we may be slower to respond to eyes than arrows in unambiguous and highly predictive contexts, the relative cost associated with disregarding non-predictive spatial information was significantly less for eyes compared with arrows. There are two potential explanations for this. First, if direct gaze activates subsequent mentalising processes (discussed above), this could slow down responsivity when the ostensive cue is not needed either because (1) there is only a single communicative gaze shift (No Search) or (2) the predictive sequence (i.e., repeated cueing of target) provides adequate information for identifying the joint attention cue (Predictive Search). However, the recruitment of mentalising mechanisms by direct gaze may *facilitate* responsivity in more ambiguous and volatile contexts by emphasising communicative intent and increasing the perceived significance of subsequent gaze behaviour (Cary, 1978; Csibra & Gergely, 2009). This is supported by findings in which seeing self-directed gaze (i.e., from a second person perspective) or observing eye contact shared between two other people (i.e., from a third person perspective) has been shown to increase gaze following and gaze cueing (Böckler et al., 2014; Bristow et al., 2007; Senju & Csibra, 2008). In this study, the relative stability in responsivity to gaze across contexts was not observed on arrow trials, where in lieu of eye contact, an arrow was temporarily replaced by a green fixation point (see Figure 2b). Unlike direct gaze, this fixation point stimulus may provide a less obvious signal of the self-relevance of subsequent spatial cues. This interpretation also aligns with findings of direct gaze increasing self-referential processing and self-involvement in social interactions (Baltazar et al., 2014; Hazem et al., 2017; Pönkänen et al., 2011), while modulating social attention and arousal (Conty et al., 2016; Senju & Johnson, 2009). It is also possible that eye contact primes participants to respond to subsequent gaze shifts by attenuating attention in a way that does not necessarily recruit higher-order mentalising representations. If so, this would also facilitate subsequent gaze responsivity irrespective of the preceding sequence of non-communicative gaze. Future work systematically manipulating the presence and duration of eye contact is needed to characterise and confirm the role of eye contact during responsive joint attention. Further work manipulating beliefs in the human agency of gaze will also elucidate whether these effects likely involve higher-order social-cognitive mechanisms (e.g., mental state attribution).

A second explanation for the relative stability in gaze responsivity across contexts is that humans have expertise in disregarding irrelevant spatial information conveyed by eyes but not arrows. The Random Search context in both experiments presented participants with the additional cognitive demand of disregarding the irrelevant and uninformative spatial information presented during the search phase. This demand was not present in the other contexts because there was either (1) no spatial information to disregard (No Search) or (2) the spatial information was informative (Predictive Search). It is therefore possible that the context effects in both experiments were larger for arrows than eye gaze because humans have daily experiences in evaluating and selectively responding to communicative eye movements while disregarding the majority of (non-communicative) eye movements that others make during face-to-face encounters. In contrast, humans may learn to almost indiscriminately orient to the spatial information conveyed by arrows, which are usually presented in isolation, as a static symbolic cue. As such, for dynamic arrows, random rather than predictive spatial sequences have a much more disruptive impact on responsivity than is seen for eyes.

### Implications

This study highlights the influence of spatio-temporal factors on responsive joint attention and the potential role of eye contact in facilitating the navigation of non-communicative and communicative spatial signals in interactive contexts. As such, this study emphasises the importance of considering these contextual factors in the ecologically valid measurement of joint attention. This is particularly relevant to studies attempting to explain why responsive joint attention may diverge in certain populations. Indeed, this study raises new possibilities for the interpretation of our previous findings with people on the autism spectrum (Caruana et al., 2018) and those diagnosed with schizophrenia (Caruana et al., 2019). In these studies, group differences in the social Random Search condition were believed to reflect differences associated with evaluating the communicative intent of gaze, given that the same differences were not observed in a non-social arrow condition. In this study, the implementation of a more precisely matched arrow version of the Random Search condition revealed that the Random Search context can result in slower response times for arrows as well as eye gaze cues. This raises the possibility that differences in responsivity to gaze-cued joint attention, previously reported in autism, could instead reflect domain-general differences in the ability to disregard irrelevant spatial information. Future work implementing our new arrow conditions—that are more closely matched to the eye gaze conditions in terms of the spatio-temporal dynamics and visual salience of the spatial cue—is needed to test this possibility.

The findings from Experiment 1 contrast with those reported in a similar study we conducted in 2017 (Caruana et al., 2017a). In both this study and that one, participants completed eye gaze and arrow versions of the Random Search and No Search task contexts. However, in the 2017 study, the non-social Random Search condition did not use arrow stimuli that “searched” during the search phase. Similar to Experiment 1 in this study, participants were slower to respond to gaze than arrows on the No Search task. In contrast to Experiment 1, this effect was even greater in the Random Search task.

These different study designs and outcomes highlight the importance of providing a good match between social and non-social stimulus conditions in terms of perceptual features and “behaviour.” However, the extent of that match raises questions about where to “draw the line” between creating valid social and non-social stimuli in studies of joint attention. Is it ecologically valid to use non-social stimuli with social behaviour if that behaviour is unlikely to be observed in natural non-social contexts? Would this dilute or eliminate the social phenomenon we are trying to index? Or would searching for the point where a non-social stimulus is no longer processed differently to a social stimulus actually provide important clues about what stimulus features make a stimulus social? Further philosophical and empirical work is needed to inform how we should design control conditions in the investigation and measurement of social-cognitive processes—particularly in interactive contexts where social information, such as gaze, is used to guide spatial attention. For instance, future research could examine whether context effects, for eyes and arrows, are influenced by whether participants believe the avatar or arrow stimuli are controlled by a human interlocutor or a computer programme (e.g., Caruana, Spirou, & Brock, 2017; Caruana & McArthur, 2019). Manipulating the attribution of mental states in this way may elucidate whether the interaction effects observed in this study are the product of higher-level social-cognitive processes recruited when we mentalise, or whether they reflect more basic effects of arousal or attention that are driven by the “socialness” of the observed stimuli.

## Conclusion

This study shows that joint attention responsivity is influenced by the presence and predictability of non-communicative eye movements made by a social partner before a joint attention bid. Critically, however, responsivity to gaze-cued joint attention is relatively stable when compared with responsivity to dynamic arrow cues in matched task contexts, where participants demonstrate significantly larger costs in response times when there is a need to parse and disregard irrelevant and non-predictive spatial information. Our data align with theories of direct gaze sensitivity which suggest that eye contact can be used as an ostensive signal to identify and selectively respond to gaze-cued joint attention, given its role in attenuating attention while increasing arousal and self-referential processing (Conty et al., 2016; Senju & Johnson, 2009). Our findings might also indicate differences in expertise acquired for gaze and arrow responsivity and attention allocation, highlighting the need for future work investigating contextual effects on gaze-following throughout development. Finally, this study identifies several issues concerning experimental design in studies of social attention. We highlight the importance of considering dynamic contexts and carefully matched non-social control conditions in the investigation of joint attention—particularly when testing whether effects are specific to the social domain of cognition. Future work investigating other contextual factors (e.g., gaze duration and temporal dynamics) of both direct and averted gaze during face-to-face interactions is needed to fully characterise how we identify, evaluate, and respond to social communicative gaze during everyday interactions.

## Supplemental Material

QJE-STD-19-379.R2-Supplementary_Material – Supplemental material for The effect of non-communicative eye movements on joint attention Supplemental material, QJE-STD-19-379.R2-Supplementary_Material for The effect of non-communicative eye movements on joint attention by Nathan Caruana, Ayeh Alhasan, Kirilee Wagner, David M Kaplan, Alexandra Woolgar and Genevieve McArthur in Quarterly Journal of Experimental Psychology

## Notes

1. For Experiment 1 (but not for Experiment 2), the saccadic reaction time (SRT) model failed to converge when including by-subject random slopes for the fixed effects. However, parameters could still be estimated. We have reported parameter estimates on the fully specified model as these estimates are more conservative than those produced by a model without random slopes. This is because the fully specified model accounts for individual differences in the effects of Stimulus and Context. Importantly, however, as we show in the accompanying analysis code and output (accessible here: https://osf.io/jb8fv/), the pattern of results does not change between these models.
