# Supplementary material for: The effect of non-communicative eye movements on joint attention
Source: Q J Exp Psychol (Hove). 2020 Aug 5;73(12):2389–402. doi: 10.1177/1747021820945604 (PMC7672778; doi:10.1177/1747021820945604)
Supplement: QJE-STD-19-379.R2-Supplementary_Material – Supplemental material for The effect of non-communicative eye movements on joint attention [file QJE-STD-19-379.R2-Supplementary_Material.docx]

Supplementary Material for:

**The effect of non-communicative eye movements on joint attention**

Caruana, N., Alhasan, A., Wagner, K.1, Kaplan, D.M.1,2, Woolgar, A2,3. & McArthur, G.

**Supplementary Material 1: Subjective Ratings Analysis and Results**

**Statistical Analysis.**

The subjective ratings were analysed using non-parametric Mann-Whitney U tests to determine if there were statistically significant effects of context (Experiment 1: Random Search versus No Search; Experiment 2: Random Search versus Predictive Search) or stimulus (eyes versus arrow). All analyses had a significance criterion of *p* < .05.

**Results**

**Experiment 1.**

We wanted to capture the subjective experiences of participants to and identify whether there were any differences between context and stimulus conditions. Figure 1 summarises the subjective task ratings for Experiment 1. The majority of participants in the final sample reported being completely convinced that their virtual partner was controlled by another person (*M* = 8.29, *SD* = 2.47). Most participants rated Alan as highly cooperative (*M* = 9.44, *SD* = 0.61). Participants also provided moderate ratings when reporting how human-like the interaction with Alan felt (*M* = 6.96, *SD* = 1.27), how human-like he behaved (*M* = 6.58, *SD* = 1.62) and appeared (*M* = 7.08, *SD* = 1.32).

**Rating comparisons by context.** No significant context effects were found for ratings of task pleasantness in either stimulus condition (Social: *W* = 57.5, *p* = .348; Non-social: *W* = 45.0, *p* = .224). No significant differences were found for ratings of how natural they found the interaction with Alan (*W* = 54.0, *p* = .271). However, participants rated the No Search condition to be significantly more natural than the Random Search condition for arrow trials (*W* = 54.0, *p* = .016). The Random Search context was also rated as more difficult than No Search context in both stimulus conditions (Eyes: *W* = 246.5, *p* < .001; Arrow: *W* = 185.5, *p* < .001).

**Rating comparisons by stimulus**. No significant differences in stimulus condition were found for pleasantness ratings for the No Search context (*W* = 103.5, *p* = .061). In contrast, eye gaze trials were reported to be significantly more pleasant than arrow trials during the Random Search context (*W* = 125, *p* = .019). Eye gaze trials were also rated as feeling more natural than arrow trials across both contexts (Random: *W* = 148.0, *p* < .001; No Search: *W* = 169, *p* = .016). Arrows were also rated as significantly more difficult than eyes in the Random Search context (*W* = 51, *p* = .042), but not in the No Search context (*W* = 79.0, *p* = .501)

**Preference rating.** Preference ratings revealed that most participants preferred face-to-face interactions than virtual interactions (*M* = 6.85, SD = 2.94). Participants also revealed a mild preference for the Random Search context despite being more difficult (M = 5.5, SD = 3.68), and preferred completing the task socially with Alan than with the arrows (*M* = 2.46, *SD* = 1.902).

| 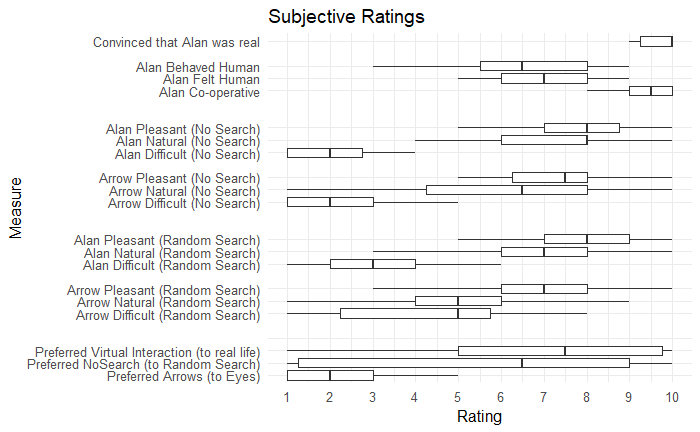 |
| --- |
| *Figure 1.* Tukey boxplots depicting responses to subjective ratings questions. |

**Experiment 2.**

As in Experiment 1, we wanted to explore whether participants’ subjective experiences differed across context and stimulus conditions. Figure 2 summarises the subjective task ratings for Experiment 2. The majority of participants reported being completely convinced that their virtual partner was controlled by another person (*M* = 8.35, *SD* = 2.66).

**Rating comparison by partner (Alan versus Tony).** Most participants found (predictive) Tony to be more cooperative than (random) Alan (*W* = 33.5, *p* = .002). Participants rated interactions with Tony as feeling more human-like (*W* = 18, *p* = .001) and his behaviour appeared more human-like (*W* = 12.0, *p* = .017).

**Rating comparisons by context.** No significant task differences were found for ratings of how pleasant participants found the different context trials across both stimulus conditions (Eyes: W = 57.5, p = 0.214; Arrow: W = 27.5, p = 0.3585). Both eyes and arrows were rated as more natural in the Predictive Search than the Random Search blocks (Eyes: *W* = 27.5, *p* = .001; Arrow: *W* = 10.5, *p* = .042). The Random Search trials were also rated as significantly more difficult than the Predictive Search trials in the arrow condition (*W* = 176.5, *p* = .006) but not the eye gaze condition (*W* = 154.5, *p* = .061).

**Rating comparisons by stimulus.** Participants reported higher pleasantness ratings for eyes than arrows for both contexts (Random: *W* = 175.0, p = .007; Predictive: *W* = 140.0, *p* = .002). They also reported that eyes felt more natural than arrows across both contexts (Random: *W* = 256.0, *p* < .001; Predictive: *W* = 262.5, *p* < .001). Also, arrows were rated as more difficult than eyes in the Random Search context (*W* = 32.5, *p* = 0.01), but not in the Predictive Search context (*W* = 64.0, *p* = .204)

**Preference rating.** Preference ratings revealed that participants varied vastly in whether they preferred having a real-life interaction or a virtual reality interaction (*M* = 5.43, *SD* = 3.57), with stronger preferences for interacting with Tony over Alan (*M* = 7.32, *SD* = 3.06), and for completing the task together with a partner (either Tony or Alan) rather than with arrows (*M* = 2.18, *SD* = 2.42).

| 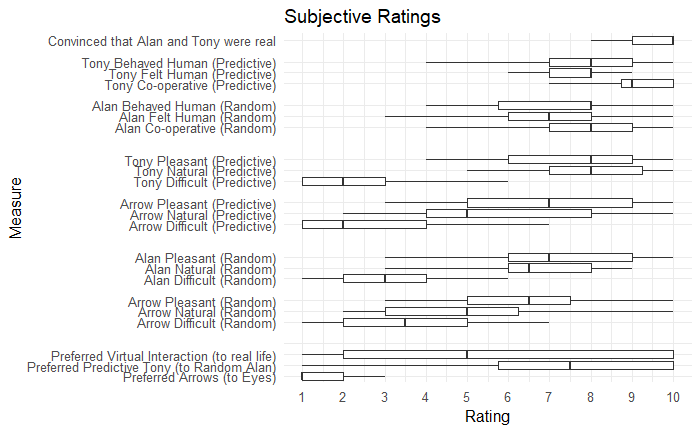 |
| --- |
| *Figure 2.* Tukey boxplots depicting responses to subjective ratings questions. |
